# Listening to Women’s Voices: The Quality of Care of Women Experiencing Severe Maternal Morbidity, in Accra, Ghana

**DOI:** 10.1371/journal.pone.0044536

**Published:** 2012-08-31

**Authors:** Özge Tunçalp, Michelle J. Hindin, Kwame Adu-Bonsaffoh, Richard Adanu

**Affiliations:** 1 Department of Population, Family and Reproductive Health, Johns Hopkins Bloomberg School of Public Health, Baltimore, Maryland, United States of America; 2 Department of Obstetrics and Gynecology, Korle-Bu Teaching Hospital, University of Ghana Medical School, College of Health Sciences, Accra, Ghana; 3 Department of Population, Family and Reproductive Health, School of Public Health, University of Ghana, Accra, Ghana; Aga Khan University, Pakistan

## Abstract

**Background:**

Women who survive severe obstetric complications can provide insight into risk factors and potential strategies for prevention of maternal morbidity as well as maternal mortality. We interviewed 32 women, in an urban facility in Ghana, who had experienced severe morbidity defined using a standardized WHO near-miss definition and identification criteria. Women provided personal accounts of their experiences of severe maternal morbidity and perceptions of the care they received.

**Methods and Findings:**

The study took place in a referral facility in urban Accra, and semi-structured interviews were conducted with women who had either a maternal near miss (n = 17) or a potentially life-threatening complication (n = 15). The most common themes surrounding the traumatic delivery were the fear of dying and concern over the potential (or actual) loss of the baby. For many women, the loss of a baby negatively influenced how they viewed and coped with this experience. Women’s perceptions of the quality of the care highlighted several key factors such as the importance of information, good communication and attitudes, and availability of human (i.e., more doctors) and physical resources (i.e., more beds, water) at the facility.

**Conclusions:**

Our results suggest that experiences of women with severe maternal morbidity may inform different aspects of quality improvement in the facilities, which in turn have a positive impact on future health seeking behavior, service utilization and reduction in maternal morbidity and mortality.

## Introduction

As part of the Millennium Development Goals, preventing the avoidable loss of women’s lives in pregnancy and childbirth in low- and middle-income countries has been a subject of extensive research and campaigning over the last two decades. [1] Still approximately 9.5 million women around the world suffer from pregnancy-related complications, and over 270,000 die. [2]–[4] Today, women in these countries are increasingly delivering at health facilities and seeking care for complications, especially in urban areas. Therefore to reduce maternal mortality and morbidity at this context, it is essential to improve the quality on the supply side of the service delivery equation. [5]

Women who survive severe obstetric complications including near miss are a vulnerable population who often suffer from both the immediate and longer-term physical, social, financial and psychological consequences of the near miss. [6] This population can provide insight into risk factors and potential strategies for prevention of maternal morbidity as well as maternal mortality, as women who experience a near miss share many characteristics with women who have died. [7], [8]

Several studies have been conducted on women’s experiences with severe maternal morbidity using different identification criteria for near miss. [1], [9], [10] Depending on the identification approach, the prevalence of near miss varies. According to a recent systematic review, prevalence rates of near miss varied between 0.6 and 14.98% for disease-specific criteria, between 0.04 and 4.54% for management-based criteria and between 0.14 and 0.92% for organ-based dysfunction based on Mantel criteria. [11] The rates are higher in low-income and middle-income countries of Asia and Africa.

This study elicits personal accounts of Ghanaian women who survived a severe maternal morbidity identified by the new WHO near-miss criteria [12] in an urban facility to explore women’s experiences of severe maternal morbidity and perceptions of the care they received to better understand quality of care. These data can help improve quality of care within the facility as well as the reputation of the facility within the community and complement the results from quantitative analyses on severe maternal morbidity and care, as the women who survived are alive to tell their stories.

### Framework

Among others, we reviewed two frameworks by Bruce (1990) [13] and Hulton et al. (2000) [14] to organize our results and explain the interconnections in women’s experiences and perceptions of quality of care. We then developed our own framework based on our data. The Bruce framework is based on improving the quality of family planning and related reproductive health care services based on six elements (i.e. choice of methods, information given to clients) leading to client-based impacts in terms of satisfaction and health outcomes. [13] The framework by Hulton et al. was specifically designed to evaluate the quality of maternity care in institutions and divided the quality into two constituent parts; the quality of provision of care within the institutions (i.e. referral system, use of appropriate technology, management of emergencies) and the quality of care experienced by the users. [14] For the purposes of this manuscript, we focus on the latter. After providing a brief overview of the women in our sample, we describe the quality of care they received organized by the following themes: provider-client information (communicating the situation and the outcomes to the patient), interpersonal relations (including attitude of the providers) and human and physical resources.

**Figure 1:**
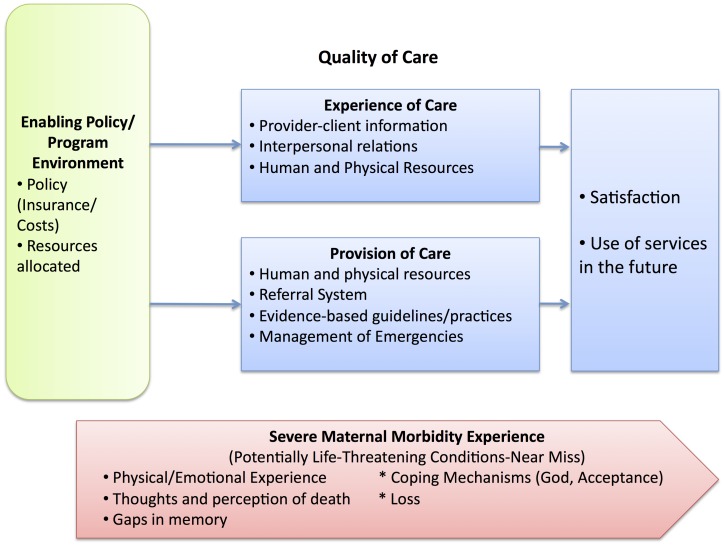
Quality of Care Framework for Severe Maternal Morbidity [13], [14].

This modified framework depicts the factors that women discussed in their experiences of severe maternal morbidity. These factors include their coping mechanisms, impact of loss of a baby, and factors important in how they perceive the care they received, which in turn may influence their satisfaction and future use of services (Figure 1). *Enabling policy/program environment* refers to the elements, which may indirectly influence both the provision and experience of care. In this case, the uptake of the national health insurance scheme and free maternity services as well as other facility-related costs impact women’s experiences. As shown in our framework, all of these elements are interrelated and influence how women cope with severe maternal morbidity and their perceptions of care.

## Methods

The study took place in Korle-Bu Teaching Hospital in urban Accra as part of a larger quantitative study on maternal near miss and quality of care, which identified all potentially life-threatening conditions, near-miss cases and maternal deaths in this process. Ghana is a West African country with a high maternal mortality. According to the latest WHO estimates, the maternal mortality ratio is 350 per 100,000 live births. [4] There is also an increasing trend in facility deliveries. Nationally, 57% of births in the five years preceding the survey occurred in health facilities and skilled health providers assisted 59% of the women during delivery. Moreover, in the Greater Accra region, where our study site is located, four out of five births occur in a health facility. [15]

The study sample was recruited by purposeful sampling from a group of women who either had a potentially life-threatening condition or maternal near miss at the facility during this period. In line with the recent WHO report, maternal near miss was defined as “*a woman who nearly died but survived a complication that occurred during pregnancy, childbirth or within 42 days of termination of pregnancy*”. [12] Identifying near-miss cases was a two-step process. First, women with potentially life-threatening conditions were identified, based on whether they had any severe complication (severe postpartum hemorrhage, severe preeclampsia, eclampsia, sepsis or severe systemic infection, ruptured uterus). Additionally, their charts were reviewed to see if they had received blood products, had a laparotomy-excluding C-section, were admitted to the ICU or spent more than six hours in the recovery room. Second, near-miss cases were identified by organ system dysfunction based on clinical criteria, laboratory markers and management-based proxies. [16]

Table 1 explains in detail the criteria used to identify potentially life-threatening conditions and near-miss cases. Data on these cases were collected from patient files and were double-checked by the study team obstetrician who was informed about the care these patients had received. The identification of these cases among the obstetric population and further details on the quantitative portion of this study are discussed in detail elsewhere. [17]

**Table 1:** Criteria to identify potentially life-threatening conditions and near miss [16].

**POTENTIALLY LIFE-THREATENING CONDITIONS**
**Severe complications**
*1. Severe postpartum hemorrhage*: genital bleeding after delivery, with at least one of the following perceived abnormal bleeding (1000 mL or more) or any bleeding with hypotension or blood transfusion.
*2. Severe preeclampsia*: Persistent systolic blood pressure of 160 mmHg or more or a diastolic blood pressure of 110 mmHg; proteinuria of 5 g or more in 24 hours; oliguria of <400 ml in 24 hours; and HELLP syndrome or pulmonary edema. Excludes eclampsia.
*3. Eclampsia:* generalized fits in a patient without previous history of epilepsy. Includes coma in preeclampsia.
*4. Sepsis or severe systemic infection*: presence of fever (body temperature >38°C), a confirmed or suspected infection (e.g. chorioamnionitis, septic abortion, endometritis, pneumonia), and at least one of the following- heart rate>100, respiratory rate>20, leukopenia (white blood cells <4000), leukocytosis (white blood cells >12 000)
*5. Ruptured uterus:* ruptured uterus during labour
**Critical Interventions**
1. Use of blood products
2. Laparotomy (including hysterectomy, excluding C-section)
3. Admission to Intensive Care Unit/recovery room > = 6 hours
**NEAR-MISS CRITERIA**
**Clinical Organ Dysfunction**
1. Acute cyanosis
2. Gasping
3. Respiratory rate >40 or <6 bpm
4. Shock
5. Cardiac Arrest
6. Oliguria non-responsive to fluids or diuretics
7. Any loss of consciousness lasting >12 hours
8. Stroke
9. Uncontrollable fit/status epilepticus
10. Global paralysis
11. Jaundice in the presence of pre-eclampsia
**Laboratory markers of organ dysfunction**
12. O_2_ saturation <90% for more than 60 min
13. PaO_2_/FiO_2_<200 mmHg
14. Creatinine>300umol/ml or >3.5 mg/dL
15. Bilirubin>100umol/L or >6.0 mg/dL
16. pH<7.1
17. Lactate >5mEq/L
18. Acute thrombocytopenia (<50,000 platelets)
**Management-based proxies**
19. Hysterectomy following infection of hemorrhage
20. Use of continuous vasoactive drugs
21. Cardio-pulmonary resuscitation
22. Dialysis for acute renal failure
23. Any non-anesthetic intubation or ventilation
24. Transfusion of >5 units of blood or red cells

In parallel with the quantitative data collection, interviews were conducted between October 2010 and March 2011. Each week, two women (one who had a potentially life-threatening condition and one who had a near miss) were randomly selected from the logbook. If the woman was discharged before the interviewers were able to approach her for invitation to the study or she refused, then another woman was randomly selected in the same category (near miss or potentially life-threatening conditions).

Interviewers invited women to participate just prior to their discharge and written informed consent was obtained from all women who agreed to participate. All study participants were asked whether they objected to the interviews being digitally recorded and none of the study participants objected. The study protocol was reviewed and approved by Johns Hopkins School of Public Health Institutional Review Board and University of Ghana Medical School College of Health Sciences.

Thirty-two women were interviewed with a semi-structured interview guide, consisting of primarily open-ended questions. Interviewers asked the participants questions in either English or Twi, depending on the woman’s preference. A trained interviewer, in a private room, interviewed the women. Prior to the interview, a short survey was administered to obtain socio-demographic data (age, parity, marital status, place of residence, education level and socioeconomic status). The interview guide included topics such as a woman’s initial expectations related to the pregnancy; her perception of the complications; the process of transfer and care; her experiences, views and opinions related to her health; the care she received; and her suggestions for improvement at the hospital. The interviews lasted between 30–45 minutes each. All of the interviews were taped and, if in Twi translated, and then transcribed in English. All of the translations and transcriptions were reviewed and edited where necessary by a second researcher before data analysis.

### Data Management and Analysis

Transcripts were analyzed and coded thematically by two researchers. The interviews were read repeatedly to identify all statements about women’s thoughts and emotions surrounding the complication and the delivery experience. We used ‘coding up’ rather than ‘coding down’, meaning the codes were developed based on the data and were not defined prior to data collection [18]. The codes were managed using hyperResearch Version 3.0.3 and all of the developed codes and themes were discussed and agreed on between the two researchers after which all transcripts were coded using the agreed-upon coding scheme.

After all of the transcripts were coded, matrices were created to help identify patterns and themes in the data, discerning differences and similarities between women with severe maternal morbidity (near miss versus potentially life-threatening conditions) and making connections broadly between the themes. The most common themes are presented.

## Results

### Study Participants

Among the 32 women interviewed, almost all of them were married, Christians and working women with at least a junior high school education. The average age was 29±4.8 and the average gestational age was 32±4.5 weeks. On average, the hospital stay was 13 days. Most women had a C-section (68.7%). A majority of the women came to the hospital as referrals (65.6%). General characteristics of these women are described in Table 2. A majority of patients had severe preeclampsia, followed by severe postpartum hemorrhage among other complications, similar to the general study population. Seventeen women were near-miss cases (56%) according to the WHO definition, and the remaining experienced severe maternal complications (potentially life-threatening conditions). In addition, fifteen women (47%) experienced a stillbirth.

**Table 2:** Socioeconomic and reproductive health indicators of the women interviewed (N = 32).

Characteristic	n (%)
**Near-Miss Status**	
*Near Miss*	17 (53)
*Potentially Life-Threatening Conditions*	15 (47)
**Reproductive Health Indicators**	
Parity, mean (range)	1.5 (0–5)
Ever Had a Miscarriage	12 (37.5)
Ever Had an Abortion	19 (59)
Ever Had a Stillbirth	11 (34.4)
Stillbirth in the current pregnancy	15 (46.8)
**Education^1^**	
None	1 (3.6)
Junior High Secondary	12 (37.5)
Senior High Secondary	12 (37.5)
Tertiary	3 (9.4)
**Occupation**	
Trader	17 (53.1)
Hairdresser/Seamstress	6 (18.8)
Other (Secretary, Housewife etc.)	9 (28.1)
**Socioeconomic Indicators**	
*Drinking Water*	20 (62.5)
Sachet water	
Piped Water	11 (34.4)
Bottled water	1 (3.1)
*Toilet Facility*	18 (56.3)
Pit latrine (Ventilated or with slab)	
Flush of piped sewer system or septic tank	14 (43.7)
*Floor Material*	
Cement	25 (78.1)
Earth/Sand	4 (12.5)
Ceramic Tile/Terrazzo	3 (9.4)
Household items, mean (range)	8.8 (2–18)
Household ownership of major items, mean (range)	0.47 (0–2)

* Four of the medical records had “unknown”.

### Accounts of Severe Maternal Morbidity

In women’s narratives, there were many references to loss of health, blood as well as feared or actual loss of life and loss of ‘normality’, which referred to feeling weak and not being able to perform tasks normally. Almost all of the women also emphasized the importance of God’s will in terms of both lives saved and lost.

Throughout the interviews, a majority of the women reported some form of negative emotion such as fear, unhappiness, hopelessness, discouragement and frustration.

*“Because I had already lost two previous pregnancies. So when this was happening, I had no hope.”* (30 years old, potentially life-threatening condition, live birth)

Some women specifically reported of being scared of an operation or the study site:

*“I was very very scared. I was afraid to the extent that I was asking myself what could have happened that I had to end up here in Korle-Bu. That [the most difficult part of the delivery experience] was the operation… For the operation, I was scared, because I never wanted to be operated in my life.”* (23 years old, potentially life-threatening condition, live birth)

Two major themes were highlighted during the interviews about the delivery experiences including thoughts and perception of death and dealing with gaps in memory.

#### Thoughts and perception of death

Many women mentioned death repeatedly throughout their interviews and discussed the impression that death was close and imminent and in some cases thinking that they were already dead.

*“I thought I would die… I could die at that time. Especially when the doctor showed the dress he was wearing… Where I was lying, I saw that that doctor’s dress was soaked with blood. And then he squeezed, is it gauze or something, and I saw the blood. I was afraid at that time that I would die.”* (37 years old, near miss, live birth)

*“I thought I was dead, but when I became conscious I realized that I wasn't feeling the abdominal pains any longer, and there was a plaster on my belly.”* (35 years old, near miss, stillbirth)

*“I don't even know how to say it. I have not seen such a thing before in my whole life. I have suffered a lot. I thought I was dead. I cried continuously for three days… I was thinking I was going to die. At the theatre I thought all was over. I was crying and begging… I cried because I was thinking I was going to die and leave my two kids behind… The thing that was happening to me, I knew I was on the path of death.”* (42 years old, near miss, stillbirth)

#### Dealing with gaps in memory

Many of the women interviewed experienced alteration in consciousness or remained under sedation for periods of time because of mechanical ventilation. Thus, they missed significant events that happened during their delivery; some women reported not knowing what happened as the most difficult part of their delivery experience.

*“When I got here I didn't see anything, I was here for four days before I became conscious… The most difficult part of the delivery was when I became unconscious for about 3 days or 4 days that I didn't know what was going on.”* (41 years old, potentially life-threatening condition, live birth)

Some women woke up to the realization of multiple operations including hysterectomy.

*“When I was given the force [induced] labor, I was able to deliver but the bleeding became the problem…. I felt dizzy and after that I didn't see anything again. When I became conscious, I was told that my womb had been removed.”* (33 years old, potentially life-threatening condition, stillbirth)

#### Dealing with loss and coping mechanisms

Loss of the baby influenced the way women experienced and coped with the severe maternal morbidity. While women with a live baby reported *“feeling happy to have a live baby”* at the end of it all, women who lost their babies reported lingering feelings of sadness and grief as well as concerns for the future of the marriage.

*Participant: I was weak after all those things had come out of me… I was out of human. It was my sister who did everything for me. I was very sad.*

*Interviewer: How do you feel now?*

*Participant: I'm fine, but sometimes (starts crying), I feel very sad.* (40 years old, near miss, stillbirth)

*“I don't feel happy at all about it, because this is my second [unsuccessful] birth and you know it is not easy. I like children and my husband too likes children a lot. So if it keeps on like this, I don't know what will happen to our marriage and I'm not happy.”* (26 years old, near miss, stillbirth)

Women’s accounts were replete with references to God. Religious faith was one of the main the main coping mechanisms reported among our study population. Many women accepted the loss as God’s will.

*“I am sad, because for you to carry pregnancy for nine months and then lose the baby at the end is very painful, but since I can't do anything about it, all I can say is thank you to God. So I told him [husband] about the death of the baby when he called last Friday, though he was worried, he couldn't do anything about it so… he said we should give thanks to God because he is the one that gives and the one that takes it away from us.”* (33 years old, near miss, stillbirth)

*“I don't think that much about it, because I know that everything is in the hands of God, there are doctors alright, but I think what ever that happens is the doing of God so I don't think about it too much.”* (32 years old, near miss, stillbirth)

### Perceptions of Quality of Care

Without exception, women reported “*being grateful*” for the care they received and underlined the fact that they believed they would not have survived if it were not for the care they received at the facility. Moreover, based on their experiences, they would consider giving birth at the facility, should they get pregnant again. However, when different elements of care were explored in depth, women raised issues about the health care staff and facility, which could be utilized to improve to quality of obstetric care.

Some were pleased with the quality of information they received from the health care staff.

*“The doctor is very good, the one who took me to the theatre. Because he told me everything, and everything he did, he wanted you to know. He would tell you that I am doing this and that and that etc., so I always say that I am happy with that.”* (32 years old, near miss, stillbirth)

In contrast, some women did not get adequate information from providers.

*“[After the hysterectomy] Oh, instantly they didn't tell me anything. So after about three days, I questioned my mother and she gave answers to my questions.”* (23 years old, potentially life-threatening condition, live birth)

Women’s accounts depicted both positive and negative encounters with staff (especially the nurses and midwives) in terms of attitudes of the staff.

*“Sometimes you will come to Korle-Bu and meet some doctor who is very good and whenever you come and meet him he will treat you nicely. There are some too when you come and meet them, you will be even scared to talk to them…”(37 years old, near miss, stillbirth)*

*“But the midwives, that's where we have problems. When you come here, they don't attend, even when you are crying and the baby's head is coming. They'd be sitting down chatting and laughing at you, I don't know why [in a very sad voice, almost crying]. This place is the best place to deliver. Because of the behavior of the midwives, that's why I decided not to come here.”(36 years old, near miss, live birth)*

Several women also mentioned the lack of physical resources such as water outages, lack of mosquito nets and delivery beds.

*“For example, come to the maternity ward, for days no water. You ask yourself, a whole maternity block. How do the women bath? And at the ward, everything you have to do there, you must use water. Blood is dropping each and every moment. But then you have no choice, you have to bear it.” (35 years old, potentially life-threatening condition, live birth)*

*“I think there should be enough beds so that everyone can get one because if someone who have just delivered should sleep on the floor then it's not nice, I don't like it.” (32 years old, near miss, stillbirth)*

Some women reported a significant delay in treatment while in the facility.

*“I was laid down for many hours without a doctor attending to me. Later on, a nurse came to me and examined me and she called a doctor. Then the doctor came in and asked me to go and do a scan. I stayed there for a long time even before I was taken to the delivery ward for the scan. I went to the delivery ward I was just lying down for so many hours. It was around 3∶30 am that another doctor found out that I've been there and I was bleeding then and later they said it's too late for me, the child is dead.”* (26 years old, near miss, stillbirth)

It should be noted that although women spoke of the reputation of the facility and prior bad facility delivery experiences, when faced with the complications, they were quick to decide to seek care at this tertiary facility.

Another issue raised by many women was the extra costs that incurred while at the facility. Even though the national health insurance scheme covered the delivery, there were many ‘hidden’ costs for drugs and other services.

*“There is another problem. So when you come here, they've been taking some small small money from you, two cedis here, four cedis there. Anything, a little thing that they do for you, even when they carry you from wherever you are to another place, they take money.”* (37 years old, near miss, live birth)

*“Sometimes when you come, they will tell you there are no medicines so they will write medicine for you to go and buy [outside the hospital], that one too is a financial problem.”* (26 years old, near miss, live birth)

## Discussion

Our study explored women’s experiences of potentially life-threatening events and maternal near miss as well as perceptions of the care they received during this period in an urban facility in Accra, Ghana. Many of the concerns raised were anticipated–fear of dying, concern over the potential (or actual) loss of the baby. Although we did not detect any differences between women with near miss and potentially life-threatening conditions in other aspects, we did observe that the women in the latter group were more likely to report the possibility of death (“*may be I will die*”), rather than the perception of imminent death described among the near-miss cases for whom the descriptions of the feelings during this period were in general more vivid. Another qualitative study on severe maternal morbidity in Brazil reports similar findings– women who survived describe a “maternal near-miss syndrome”, characterized by triggering components such as a potentially fatal complication with the main component of feeling as the impending death and fear. [10]

Previous research suggests that the trauma of the severe maternal morbidity is modified by the loss of the baby. [19], [20] For many women in our study, the loss of a baby negatively influenced how they viewed and coped with the traumatic delivery experience. These women were more fragile and more frequently reported negative emotions. Although our study was conducted at the hospital within the first two weeks postpartum, a longitudinal study in Burkina Faso indicated that after hospital discharge the women with severe obstetric complications had worse mental-health outcomes in the first three months after birth compared to women with uncomplicated pregnancies. [6] These results underline the vulnerability of this population and the extra attention required for the postpartum visits as well as counseling for women with stillbirth and severe maternal morbidity since they are more likely to experience postpartum morbidity. [6], [10], [21] As seen in some other African countries [9], many women spoke in religious terms about God, and God’s will was frequently mentioned to explain what had happened and also as a coping mechanism to move on with their lives.

To conceptualize our results, we used a quality of care framework from the severe maternal morbidity perspective, informed by Bruce [13] and Hulton et al [14] as well as our own data. Maternal morbidity is a spectrum between non-complicated pregnancies and maternal death. At the end of the spectrum, a woman with a life-threatening condition will become either a maternal near miss or a maternal death and in many cases there is a thin line between these two categories. In the latter category, the women are alive to share their experiences and perspectives. Studies have shown that health systems factors, service delivery and the interpersonal aspects of care play an important role and that poor quality health care services compromise access, effectiveness and compliance among other things. [22]–[24]

In our study, women’s perceptions of the quality of the care highlighted several key factors which influenced women’s perceptions, such as the importance of information, good communication, attitude, and availability of human (i.e. more doctors) and physical resources (i.e. more beds) at the facility. Good quality care should include information provided to the women about their conditions and the treatment protocols. Women should have the opportunity to ask questions and receive clear answers. However for many women in our study, this was not possible since for some period of time they were not conscious; yet, it is very important to highlight the fact that when it did occur, women reported it as calming their worries and making them feel safer in the situation. The negative attitude of the nurses and midwives were brought up by many women, which is in agreement with previous research from Africa both among general obstetric populations and among women with severe maternal morbidity. [9], [22], [24] Positive interactions in terms of communication and attitude between patients and health care providers would increase not only quality of care perceived by these women, but also improve future health care seeking for these women and their families. Also it empowers them by providing the essential information surrounding their delivery, which might be otherwise missed. This may make it easier to accept the consequences, cope with the trauma and the possible loss and move on with their lives.

Unlike the factors directly related to the health care providers, some of the key issues raised by the women are not fully within the control of the health care providers. Issues related to the lack of human and physical resources and the cost of services are such examples. Treatment at the facility was delayed in certain cases, mostly involving emergency surgeries, due to the high load of cases and unavailability of the operating room. Provision of mosquito nets, availability of water and beds for women influenced women’s experiences especially after such a traumatic delivery. [7], [9], [24] Cost was another issue commonly discussed in the interviews. The national health insurance scheme in Ghana, which started in 2003 and became fully functional in 2005, covers delivery services for women. [25], [26] But in the event of a severe maternal morbidity, the costs of hospital charges escalate rapidly and women need to pay certain expenses out of pocket, such as certain drugs not covered under the insurance or not available at the hospital pharmacy. Moreover there are small, but when added up, sizable fees these women have to bear at the facility. [7], [24] After a near-miss event, women in our study population spent on average 15 days at the ward, most of the time by themselves with limited financial resources. Usually the husbands or the families do not frequently visit the women at the facility [27] and come to pay the bills around the discharge, yet these small amounts are paid as they incur, causing additional distress for women.

Our semi-structured interviews allow for a deeper understanding of women’s severe morbidity experiences and perceptions of care compared with a review of medical records alone. By listening to women’s own voices and stories, modifiable facility level concerns that could be readily addressed can be identified. A key limitation of this study is that we do not have data from the women who died in terms of their experiences of quality of care. In addition, women who had substantial fears or the greatest concerns about the facility are likely omitted from our sample as these fears likely prevented them from seeking care. Even though we tried to interview women as close to discharge as possible, as all interviews were conducted in the hospital, women may have been reluctant to criticize the facility and/or staff while still in the facility, potentially leading to more positive reports. Also, we did not collect information about the women’s support systems, which might have influenced their perceptions accordingly.

In the quest to improve obstetric care and reduce preventable maternal morbidity and mortality, it is crucial to include the perspectives of the health-care consumers, in this case the women with potentially life-threatening complications or near miss. Although an effectively functioning referral system and a technically competent medical team who can accurately and timely identify the complications and uses evidence-based treatment protocols are essential for quality improvement [16], [28], it is obvious from the women’s accounts that good interpersonal skills and communication play a very important role in coping with severe morbidity and patient satisfaction.

Women who survive a near-miss event or severe maternal morbidity are a vulnerable population. Tertiary care hospitals are frequently perceived as places where women go to die, as word on maternal deaths and fetal loss spread quickly through communities [29]. Women often fear going to tertiary care hospitals and may face other barriers such as costs or partner opposition. Our study shows that while women had “*heard*” things about Korle Bu, given their desperation, they often came without much delay. While there were important concerns about the quality of care, most women in our sample would be willing or even prefer to deliver at Korle Bu in the future. While encouraging, more work needs to be done to improve the reputation of tertiary care hospitals so that women do not delay in seeking care. It is also important to emphasize that obstetric care does not exist in a vacuum with only the health care providers and the patients; rather it exists within the health systems environment where enabling policy and program environment such as effectively allocated resources and financial policies allowing access to affordable care make an impact in how women experience near-miss events and perceive care.

## References

[1] Storeng KT, Murray SF, Akoum MS, Ouattara F, Filippi V (2010) Beyond body counts: a qualitative study of lives and loss in Burkina Faso after ‘near-miss’ obstetric complications. Soc Sci Med 71: 1749–1756. 20541307 10.1016/j.socscimed.2010.03.056

[2] Lozano R, Wang H, Foreman KJ, Rajaratnam JK, Naghavi M, et al (2011) Progress towards Millennium Development Goals 4 and 5 on maternal and child mortality: an updated systematic analysis. Lancet 378: 1139–1165. 21937100 10.1016/S0140-6736(11)61337-8

[3] World Health Organization (2004) Beyond the numbers: reviewing maternal deaths and complications to make pregnancy safer. Geneva, Switzerland: World Health Organization.

[4] World Health Organization, UNICEF, UNFPA, The World Bank (2012) Trends in maternal mortality: 1990 to 2010. WHO, UNICEF, UNFPA, and The World Bank Estimates.

[5] Wang W, Alva S, Wang S, Fort A (2011) Levels and Trends in the Use of Maternal Health Services in Developing Countries. Calverton, Maryland, USA: ICF Macro.

[6] Filippi V, Ganaba R, Baggaley RF, Marshall T, Storeng KT, et al (2007) Health of women after severe obstetric complications in Burkina Faso: a longitudinal study. Lancet 370: 1329–1337. 17933647 10.1016/S0140-6736(07)61574-8

[7] Borghi J, Hanson K, Acquah CA, Ekanmian G, Filippi V, et al (2003) Costs of near-miss obstetric complications for women and their families in Benin and Ghana. Health Policy Plan 18: 383–390. 14654514 10.1093/heapol/czg046

[8] Pattinson RC, Hall M (2003) Near misses: a useful adjunct to maternal death enquiries. Br Med Bull 67: 231–243. 14711767 10.1093/bmb/ldg007

[9] Weeks A, Lavender T, Nazziwa E, Mirembe F (2005) Personal accounts of 'near-miss' maternal mortalities in Kampala, Uganda. BJOG 112: 1302–1307. 16101612 10.1111/j.1471-0528.2005.00703.x

[10] Souza JP, Cecatti JG, Parpinelli MA, Krupa F, Osis MJ (2009) An emerging "maternal near-miss syndrome": narratives of women who almost died during pregnancy and childbirth. Birth 36: 149–158. 19489809 10.1111/j.1523-536X.2009.00313.x

[11] Tuncalp O, Hindin M, Souza J, Chou D, Say L (2012) The prevalence of maternal near miss: a systematic review. BJOG 119: 653–661. 22489760 10.1111/j.1471-0528.2012.03294.x

[12] Say L, Souza JP, Pattinson RC (2009) Maternal near miss–towards a standard tool for monitoring quality of maternal health care. Best Pract Res Clin Obstet Gynaecol 23: 287–296. 19303368 10.1016/j.bpobgyn.2009.01.007

[13] Bruce J (1990) Fundamental elements of the quality of care: a simple framework. Stud Fam Plann 21: 61–91. 2191476

[14] Hulton L, Matthews Z, Stones W (2000) A framework for evaluation of quality of care in maternity services. Southampton: University of Southampton.

[15] Ghana Statistical Service (GSS), Ghana Health Service (GHS), ICF Macro (2009) Ghana Demographic and Health Survey 2008. Accra, Ghana: GSS, GHS, and ICF Macro.

[16] World Health Organization, (2011) Evaluating the quality of care for severe pregnancy complications: the WHO near-miss approach for maternal health. Geneva: WHO.

[17] Tunçalp Ö (2012) Severe Maternal Complications, Near Miss, and Quality of Care. Baltimore, MD: Johns Hopkins University.

[18] Keenan KF, Teijlingen Ev, Pitchforth E (2005) The analysis of qualitative research data in family planning and reproductive health care. The Journal of Family Planning and Reproductive Health Care 31: 40–43. 15720849 10.1783/0000000052972825

[19] Fottrell E, Kanhonou L, Goufodji S, Behague DP, Marshall T, et al (2010) Risk of psychological distress following severe obstetric complications in Benin: the role of economics, physical health and spousal abuse. Br J Psychiatry 196: 18–25. 20044654 10.1192/bjp.bp.108.062489 PMC2802511

[20] Saurel-Cubizolles MJ, Romito P, Lelong N, Ancel PY (2000) Women's health after childbirth: a longitudinal study in France and Italy. BJOG 107: 1202–1209. 11028569 10.1111/j.1471-0528.2000.tb11608.x

[21] Ayers S (2007) Thoughts and emotions during traumatic birth: a qualitative study. Birth 34: 253–263. 17718876 10.1111/j.1523-536X.2007.00178.x

[22] Jewkes R, Abrahams N, Mvo Z (1998) Why do nurses abuse patients? Reflections from South African obstetric services. Soc Sci Med 47: 1781–1795. 9877348 10.1016/s0277-9536(98)00240-8

[23] Gilson L, Alilio M, Heggenhougen K (1994) Community satisfaction with primary health care services: an evaluation undertaken in the Morogoro region of Tanzania. Soc Sci Med 39: 767–780. 7973873 10.1016/0277-9536(94)90038-8

[24] D'Ambruoso L, Abbey M, Hussein J (2005) Please understand when I cry out in pain: women's accounts of maternity services during labour and delivery in Ghana. BMC Public Health 5: 140. 16372911 10.1186/1471-2458-5-140 PMC1343547

[25] Witter S, Garshong B (2009) Something old or something new? Social health insurance in Ghana. BMC Int Health Hum Rights 9: 20. 19715583 10.1186/1472-698X-9-20 PMC2739838

[26] Witter S, Arhinful DK, Kusi A, Zakariah-Akoto S (2007) The experience of Ghana in implementing a user fee exemption policy to provide free delivery care. Reprod Health Matters 15: 61–71. 17938071 10.1016/S0968-8080(07)30325-X

[27] Schwandt H, Creanga A, Danso KA, Adanu RM, Agbenyega T, et al. (2012) Unsafe Abortion in Ghana: The Roles of Male Partners, Women, and Health Care Providers. Under Review. 10.1016/j.contraception.2013.03.010 23643156

[28] Gulmezoglu AM (2003) Promoting standards for quality of maternal health care. Br Med Bull 67: 73–83. 14711755 10.1093/bmb/ldg001

[29] D'Ambruoso L, Byass P, Qomariyah SN, Ouedraogo M (2012) A lost cause? Extending verbal autopsy to investigate biomedical and socio-cultural causes of maternal death in Burkina Faso and Indonesia. Soc Sci Med 71: 1728–1738. 10.1016/j.socscimed.2010.05.023 20646807

